# Spatially organized macrophage–T-cell crosstalk in cervical cancer: insights from single-cell and spatial omics

**DOI:** 10.3389/fimmu.2026.1896004

**Published:** 2026-07-15

**Authors:** Li Huang, Nan Lin, Hao Li, Yong Hu

**Affiliations:** 1 Department of Oncology, Guiyang Public Health Clinical Center, Guiyang, China; 2 Department of Oncology, Guiyang Cancer Hospital, Guiyang, China; 3 Department of Oncology, Guiyang Fifth People’s Hospital, Guiyang, Guizhou, China

**Keywords:** cervical cancer, HPV, macrophage-T cell crosstalk, spatial immune niches, spatial transcriptomics, SPP1 macrophages, tumor immune microenvironment

## Abstract

Immunotherapy has transformed the therapeutic landscape of advanced cervical cancer, yet clinical benefit remains limited by a highly heterogeneous and immunosuppressive tumor microenvironment. Traditional paradigms, including binary M1/M2 macrophage polarization and models that interpret T-cell dysfunction solely through checkpoint expression, are insufficient to capture the localized intercellular dynamics that drive immune evasion. Recent advances in single-cell and spatial multi-omics have fundamentally reshaped our understanding of this landscape. In this review, we synthesize emerging high-dimensional atlases to reframe macrophage–T-cell crosstalk from simple ligand–receptor interactions into a spatially organized ecological model. We highlight the paradigm shift toward highly resolved myeloid programs, particularly SPP1^+^ and C1QC^+^ macrophage states, and discuss how these programs interact with stromal barriers, regulatory T cells, and metabolic checkpoints to restrict, exclude, or functionally constrain effector T cells within suppressive niches. Crucially, we position persistent high-risk human papillomavirus infection not merely as an initiating carcinogenic trigger, but as an upstream and continuous programmer that rewires innate immune sensing, including context-dependent cGAS–STING-related circuits, to stabilize local immune tolerance throughout disease progression. Finally, we propose translational strategies for distilling complex multi-omic atlases into pathology-compatible prognostic and predictive biomarker signatures. Ultimately, by deciphering these spatially organized networks, this review aims to provide actionable translational insights for targeting macrophage vulnerabilities, guiding biomarker-driven combinatorial immunotherapies, and overcoming immune resistance in cervical cancer.

## 1 Introduction

### 1.1 Clinical challenge: immune-context-dependent resistance in HPV-associated cervical cancer

Cervical cancer remains a major global health burden and one of the leading causes of cancer-related morbidity and mortality among women worldwide (1). Treatment options for locally advanced, recurrent, and metastatic disease have expanded in recent years, including immune checkpoint blockade, pembrolizumab-based chemoradiotherapy or chemoimmunotherapy, tissue factor-directed antibody–drug conjugates such as tisotumab vedotin, and bispecific antibody-based strategies such as cadonilimab (2–9). Nevertheless, durable benefit remains limited to a subset of patients, and both primary and acquired resistance are frequently observed (2, 3, 6, 10). These observations suggest that therapeutic failure is shaped not only by tumor-intrinsic alterations, but also by the immune context in which cervical tumors evolve.

Persistent high-risk human papillomavirus infection provides the defining biological background of cervical cancer. HPV E6/E7-driven transformation can perturb antigen presentation, interferon signaling, epithelial inflammatory programs, and local immune tolerance, creating a paradoxical setting in which viral antigenicity coexists with progressive immune dysfunction (10–17). A central unresolved question is therefore how an immunogenic virus-associated tumor becomes organized into a therapeutically resistant and immunosuppressive microenvironment.

### 1.2 Conceptual shift: from averaged immune infiltration to cell-state and spatially resolved programs

Traditional immune profiling has provided important information about the cervical cancer tumor immune microenvironment, but population-averaged measurements cannot fully resolve rare cell states, developmental trajectories, or spatially restricted cell–cell interactions (18–22). This limitation is particularly relevant for tumor-associated macrophages. Although the M1/M2 framework remains historically useful, recent single-cell studies indicate that human TAMs are better understood as context-dependent transcriptional programs rather than fixed polarization states (23–30). These programs include inflammatory, interferon-responsive, antigen-presenting, lipid-metabolic, tissue-remodeling, SPP1-associated, and TREM2-expressing macrophage states that are not reliably captured by conventional polarization markers alone (24–30).

A similar refinement is needed for T cells. CD8^+^ T-cell abundance does not necessarily indicate effective antitumor immunity, because tumor-infiltrating T cells may become bystander-like, dysfunctional, or terminally exhausted within hypoxic, metabolically stressed, and myeloid-enriched niches (31–37). These states are often associated with TOX, PDCD1, HAVCR2, LAG3, TIGIT, and other inhibitory or exhaustion-related modules (31, 33, 35, 36). Thus, immune infiltration in cervical cancer should be interpreted not only by abundance, but also by cell state, trajectory, clonality, and spatial localization (19–21, 31, 32, 38–45).

### 1.3 Scope of this review: spatial macrophage–T-cell crosstalk and clinically testable signatures

Single-cell and spatial omics now provide complementary approaches for dissecting cellular heterogeneity and tissue organization in cervical cancer (18–22, 32, 38–50). Single-cell RNA sequencing resolves immune and stromal cell states, whereas spatial transcriptomic and imaging-based methods preserve their localization within tumor nests, stromal regions, invasive margins, and other functional niches. However, spatially inferred communication remains hypothesis-generating and requires orthogonal validation using approaches such as immunohistochemistry, multiplex immunofluorescence, *in situ* hybridization, spatial proteomics, or functional perturbation assays (19, 21, 22, 50).

Recent studies have begun to reveal how suppressive macrophage programs, dysfunctional T-cell states, fibroblast-associated immune exclusion, and HPV-driven immune pressure are organized across cervical cancer tissue niches, disease stages, and histological subtypes (32–34, 38–45, 47, 48, 51). Unlike reviews that discuss HPV immune escape, TAM biology, or T-cell exhaustion separately, this review places macrophage–T-cell crosstalk at the center of a spatially organized model of cervical cancer immune suppression. We focus on three interconnected themes: TAM heterogeneity beyond the M1/M2 framework, the emergence of T-cell dysfunction and exhaustion, and the spatial mechanisms that couple macrophage and T-cell compartments. Finally, we discuss how recurrent atlas-derived signatures may be distilled into FFPE-compatible biomarker panels for prognostic stratification, response prediction, and biomarker-enriched trial design (21, 43, 45, 49–52).

## 2 Reframing TAM heterogeneity in cervical cancer: from M1/M2 polarization to macrophage programs

The classical M1/M2 framework remains a useful historical reference, but it is insufficient to capture the diversity of tumor-associated macrophage states revealed by single-cell and spatial studies (23, 26–28). In cervical cancer, TAMs are better viewed as dynamic and partially overlapping transcriptional programs shaped by HPV-associated inflammation, tumor progression, stromal architecture, hypoxia, and local immune pressure (11, 32, 38–43, 45–48, 51, 53, 54). This section summarizes recurrent TAM programs in cervical cancer, with emphasis on SPP1-associated, C1Q-enriched, and TREM2/APOE-associated states and their potential relevance to immunosuppressive niches (24, 25, 29, 30, 32, 43, 45, 51).

### 2.1 Recurrent TAM programs identified by single-cell atlases

Single-cell atlases have replaced the binary M1/M2 model with a multidimensional view of macrophage heterogeneity. Across tumor types, including cervical cancer, TAMs span inflammatory, interferon-responsive, antigen-presenting, complement-associated, lipid-metabolic, angiogenic, and matrix-remodeling programs rather than discrete polarization endpoints (24, 25, 28, 30, 32, 38–43, 45–48, 51). In cervical cancer, these programs should be interpreted within a virally conditioned mucosal context, where persistent HPV exposure, epithelial transformation, and chronic epithelial–stromal inflammation may jointly influence myeloid recruitment and macrophage education (11–17, 38–42, 46–48) (**Table 1**).

**Table 1:** Recurrent TAM programs in cervical cancer and related tumor contexts.

TAM program	Representative markers	Putative functions	Evidence in cervical cancer	Key caution
SPP1-associated TAM	SPP1, CD44-related signaling, VEGFA, MMPs	ECM remodeling, angiogenesis, immune suppression	Recurrently detected in cervical cancer atlases	Mostly association; causal validation needed
C1Q-enriched TAM	C1QA, C1QB, C1QC	Complement activity, phagocytosis, tissue remodeling	Prognostic and heterogeneity-related signals reported	Not always suppressive
TREM2/APOE TAM	TREM2, APOE, LPL, lipid genes	Lipid metabolism, phagocytic remodeling, immunoregulation	Strong pan-cancer evidence; limited cervical spatial validation	Needs cervical-specific validation
Hypoxia-conditioned TAM	HIF1A-related genes, VEGFA, CXCLs/CCLs	Recruitment, angiogenesis, metastasis	Supported by ZEB1–CCL8 and CD47–SIRPα studies	Mechanistic links require spatial confirmation

Among these states, SPP1-associated TAMs are one of the most recurrently identified programs. They typically express SPP1 together with genes related to angiogenesis, extracellular matrix remodeling, hypoxia adaptation, and tissue repair, suggesting roles in invasive progression and local immune suppression (25, 28, 32, 43, 45, 51, 55). Cervical cancer atlases indicate that SPP1^+^ macrophages are enriched in malignant lesions and may communicate with tumor, stromal, and immune compartments, frequently through the SPP1–CD44 axis (25, 32, 38, 42, 43, 48, 51, 56). Their spatial proximity to dysfunctional lymphoid compartments supports their potential involvement in suppressive niches, although direct causal evidence remains limited (25, 32, 38, 42, 43, 45, 48, 51).

C1Q-enriched and TREM2/APOE-associated macrophage programs also warrant attention. C1Q-high macrophages, marked by C1QA, C1QB, and C1QC, have been observed across tumor types and may reflect complement activity, phagocytosis, tissue remodeling, or immunoregulation depending on context (24, 30, 51). In cervical cancer, C1Q-related signatures have been linked to macrophage heterogeneity and potential prognostic relevance, but they should not be interpreted as uniformly suppressive (30, 51). TREM2/APOE-associated TAMs, characterized by TREM2, APOE, LPL, and lipid-metabolic genes, have emerged in multiple cancers as immunoregulatory macrophages involved in lipid handling, efferocytosis, phagocytic remodeling, and suppression of antitumor immunity (24, 28–30). Whether TREM2^+^ TAMs form a stable and spatially defined population in cervical cancer remains insufficiently characterized and requires disease-specific validation (32, 38–43, 48).

Together, these findings suggest that SPP1-associated, C1Q-enriched, and TREM2/APOE-associated programs represent partially overlapping macrophage axes rather than rigid cell types. SPP1^+^ TAMs are often linked to matrix remodeling, angiogenesis, and invasive niches; C1Q-enriched macrophages may reflect complement-associated phagocytic or tissue-resident-like functions; and TREM2/APOE macrophages are frequently associated with lipid metabolism and immunoregulatory remodeling. Whether these axes correspond to stable subsets, transitional states, or context-dependent transcriptional modules in cervical cancer remains unresolved.

### 2.2 Microenvironmental conditioning and inferred developmental trajectories of TAMs

TAMs should not be viewed as static residents of the tumor bed, but as dynamically reprogrammed populations shaped by sustained microenvironmental pressure (23, 26–28). In HPV-associated cervical carcinogenesis, persistent viral infection, epithelial transformation, altered interferon signaling, inflammatory cytokines, hypoxia, stromal remodeling, and tumor-derived metabolites may collectively influence macrophage recruitment and state transitions (11–17, 38, 42, 48). These inputs can divert recruited monocytes, as well as existing local macrophage populations, toward inflammatory/interferon-responsive, antigen-presenting, SPP1^+^ tissue-remodeling, or lipid-metabolic/TREM2-like immunoregulatory programs (23, 26–28, 37, 45, 53–55, 57).

Trajectory analyses in cervical cancer, together with cross-tissue monocyte/macrophage compendia, support a model in which many TAMs may arise from recruited peripheral monocytes (24, 30, 32, 38–43, 46–48, 51). However, this monocyte-centric perspective must be contextualized within the unique biology of the cervical mucosa, which inherently maintains distinct tissue-resident macrophage populations under homeostatic conditions. Currently, it remains unresolved in human cervical cancer whether these resident mucosal populations are displaced, reprogrammed, or primarily supplemented by monocyte-derived cells during HPV-driven carcinogenesis. As observed in other mucosal malignancies, tissue-resident macrophages can contribute substantially to the suppressive TAM pool and may respond quite differently to microenvironmental cues than newly recruited cells. For the monocyte-derived compartment, after entering the lesion, these cells may be progressively conditioned by niche-specific cues. Hypoxia is one example: mechanistic studies have shown that hypoxia promotes macrophage recruitment through the ZEB1–CCL8 axis and can strengthen the CD47–SIRPα innate immune checkpoint, thereby limiting macrophage-mediated phagocytosis of cervical squamous carcinoma cells (53, 54). These observations support the view that macrophage remodeling during cervical carcinogenesis reflects disease-associated reprogramming rather than simple quantitative accumulation (32, 38, 40, 42, 43, 48, 49).

However, inferred trajectories should be interpreted cautiously. Pseudotime and RNA velocity analyses suggest possible transcriptional transitions but do not establish definitive lineage relationships. TAM development in cervical cancer is therefore unlikely to follow a single linear path; instead, it probably reflects branching and context-dependent reprogramming reinforced by stromal architecture, vascular status, metabolic stress, and immune pressure (23, 26–28, 32, 37–43, 45–48, 51). Establishing true developmental relationships—and robustly distinguishing the relative contributions of tissue-resident versus recruited monocyte-derived TAMs—will require integration of single-cell transcriptomics with epigenomic profiling, spatial localization, clonal or fate-mapping approaches, and functional validation (18–22, 38–43, 45–48, 50).

### 2.3 Spatial embedding of TAM programs

Spatially resolved profiling adds an important layer to TAM biology by linking macrophage states to tissue neighborhoods (19–22, 38–42, 48). In cervical cancer, TAM programs are unlikely to be distributed uniformly across the tumor bed. Suppressive or tissue-remodeling macrophage states may be enriched in hypoxic tumor cores, invasive margins, fibroblast-rich stromal regions, aberrant vascular areas, and epithelial–stromal interfaces (32, 38–43, 45, 48, 51, 53, 54). These niches can impose distinct pressures: hypoxia may favor angiogenic and SPP1-associated remodeling programs, invasive fronts may enrich macrophages involved in matrix degradation, and fibroblast-rich regions may stabilize macrophage states associated with T-cell exclusion.

The biological significance of a TAM program therefore depends not only on its transcriptional identity, but also on its location and neighboring cell states. The same macrophage-associated program may have different consequences depending on whether it is positioned near cytotoxic T cells, exhausted T cells, regulatory T cells, fibroblast-rich stromal barriers, angiogenic tumor fronts, or hypoxic tumor cores (32, 34, 37–45, 48, 58). Candidate axes such as SPP1–CD44, CSF1–CSF1R, CCL/CCR, CXCL/CXCR, TGFB, IL10, VEGFA, and checkpoint-related interactions may contribute to these neighborhoods, but spatial proximity and ligand–receptor inference should be regarded as hypothesis-generating until validated by multiplex imaging, perturbation experiments, and clinically annotated cohorts (19, 21, 22, 50).

Overall, recent studies support a reframing of cervical cancer TAM biology away from fixed M1/M2 polarization and toward a dynamic, microenvironment-dependent, and spatially embedded model of macrophage function (23, 26–28, 32, 38–43, 45–48, 51). Within this framework, SPP1-associated, C1Q-enriched, and TREM2/APOE-associated programs appear more informative than conventional polarization labels for understanding how macrophages contribute to immune evasion, stromal remodeling, and disease progression (24, 25, 28–30, 32, 43, 45, 51, 55, 57). This macrophage-centered view provides the basis for examining how T-cell dysfunction emerges and becomes spatially coupled to suppressive myeloid niches.

## 3 Decoding T-cell exhaustion and dysfunction in cervical cancer

T-cell dysfunction is a major driver of immune escape in cervical cancer and is closely linked to suppressive myeloid, stromal, and tumor-cell programs (31–37, 43–45, 58–63). Rather than being simply “functional” or “non-functional,” tumor-infiltrating T cells occupy a continuum of transcriptional, clonal, metabolic, and spatial states shaped by persistent HPV antigen exposure, chronic mucosal inflammation, tumor-derived cytokines, metabolic stress, and suppressive cues from TAMs, Tregs, CAFs, and malignant epithelial cells (31–38, 42–45, 58–63). This issue is particularly relevant in HPV-associated cervical cancer, where viral antigens such as E6 and E7 provide immunogenic targets but may also contribute to chronic stimulation-associated dysfunction.

Compared with bulk sequencing, scRNA-seq, scTCR-seq, and spatial profiling enable simultaneous assessment of T-cell functional states, clonal architecture, and tissue localization (18–22, 31, 35, 36, 50, 59–63). These approaches have shifted the interpretation of T-cell dysfunction from a purely cell-intrinsic exhaustion model toward an ecological model in which dysfunctional T-cell states are stabilized by TAM-rich, Treg-enriched, stromal-restricted, and metabolically hostile neighborhoods. This framework helps explain why immunotherapy responses in cervical cancer remain heterogeneous despite the presence of tumor-infiltrating lymphocytes and viral tumor antigens (2–4, 7, 35, 36, 52, 59–64).

### 3.1 CD8^+^ T-cell state transitions: activation, exhaustion, proliferation, and senescence

Single-cell studies show that tumor-infiltrating CD8^+^ T cells in cervical cancer do not converge on a single terminal phenotype. Instead, they span precursor-like, migratory, cytotoxic-like, proliferative, exhaustion-like, terminally dysfunctional, and therapy-induced senescent states (31, 35, 36, 59, 62, 63). Pseudotime analysis has identified CD8^+^ T-cell subsets characterized by CXCR4-, GZMK-, and CX3CR1-associated precursor or cytotoxic-like programs, PDCD1-associated exhaustion-like programs, and MKI67-associated proliferative programs, with precursor-like cells positioned earlier and PDCD1^+^ or MKI67^+^ cells positioned later along inferred trajectories (35). Subsequent multiomic studies further supported cytotoxic decline and expansion of exhausted or dysfunctional NK/T-like states in cervical tumors (59), while integrative analyses refined CD8^+^ T cells into progenitor, intermediate, proliferative, and terminally differentiated subsets (36).

At the molecular level, exhaustion-like CD8^+^ T cells often co-express inhibitory receptors such as PDCD1, TIGIT, HAVCR2, and LAG3 (31, 33, 35, 36, 59). However, PDCD1 expression alone should not be equated with terminal exhaustion, because it may also reflect recent activation or antigen encounter. More reliable interpretation requires integration of inhibitory receptor co-expression, transcriptional regulators such as TOX or NR4A family members, cytotoxic gene expression, proliferative status, clonal expansion, and spatial localization. Markers including TCF7, IL7R, GZMK, CX3CR1, MKI67, and TOX may help distinguish precursor-like, cytotoxic-like, proliferative, transitional, and terminally dysfunctional compartments, although their meaning remains context-dependent (31, 33, 35, 36, 59) (**Table 2**).

**Table 2:** Major T-cell states and dysfunction programs in cervical cancer.

T-cell state/program	Representative markers	Functional implication	Key uncertainty
Precursor-like CD8^+^ T cells	TCF7, IL7R, CCR7, CXCR4	Potential reservoir for response to immune checkpoint blockade	Cervical cancer-specific marker combinations require harmonization
Cytotoxic-like CD8^+^ T cells	GZMB, PRF1, NKG7, CX3CR1	Direct tumor killing and effector function	May be spatially excluded or metabolically constrained
Proliferative CD8^+^ T cells	MKI67, TOP2A	Clonal activation, expansion, or proliferative exhaustion-like state	Effector versus dysfunctional proliferation requires TCR and spatial linkage
Terminally dysfunctional/exhaustion-like CD8^+^ T cells	PDCD1, TIGIT, HAVCR2, LAG3, TOX	Reduced effector plasticity and checkpoint dependence	Reversibility varies by transcriptional and epigenetic state
Senescent CD8^+^ T cells	KLRG1, senescence/stress-associated genes	Therapy-associated functional impairment	Distinct from exhaustion; upstream mechanisms require validation
Tregs	FOXP3, IL2RA, CTLA4, TIGIT, ICOS, TNFRSF4	Suppression of effector T-cell activation and expansion	Subtype- and location-specific effects remain incompletely defined

**Figure 1:**
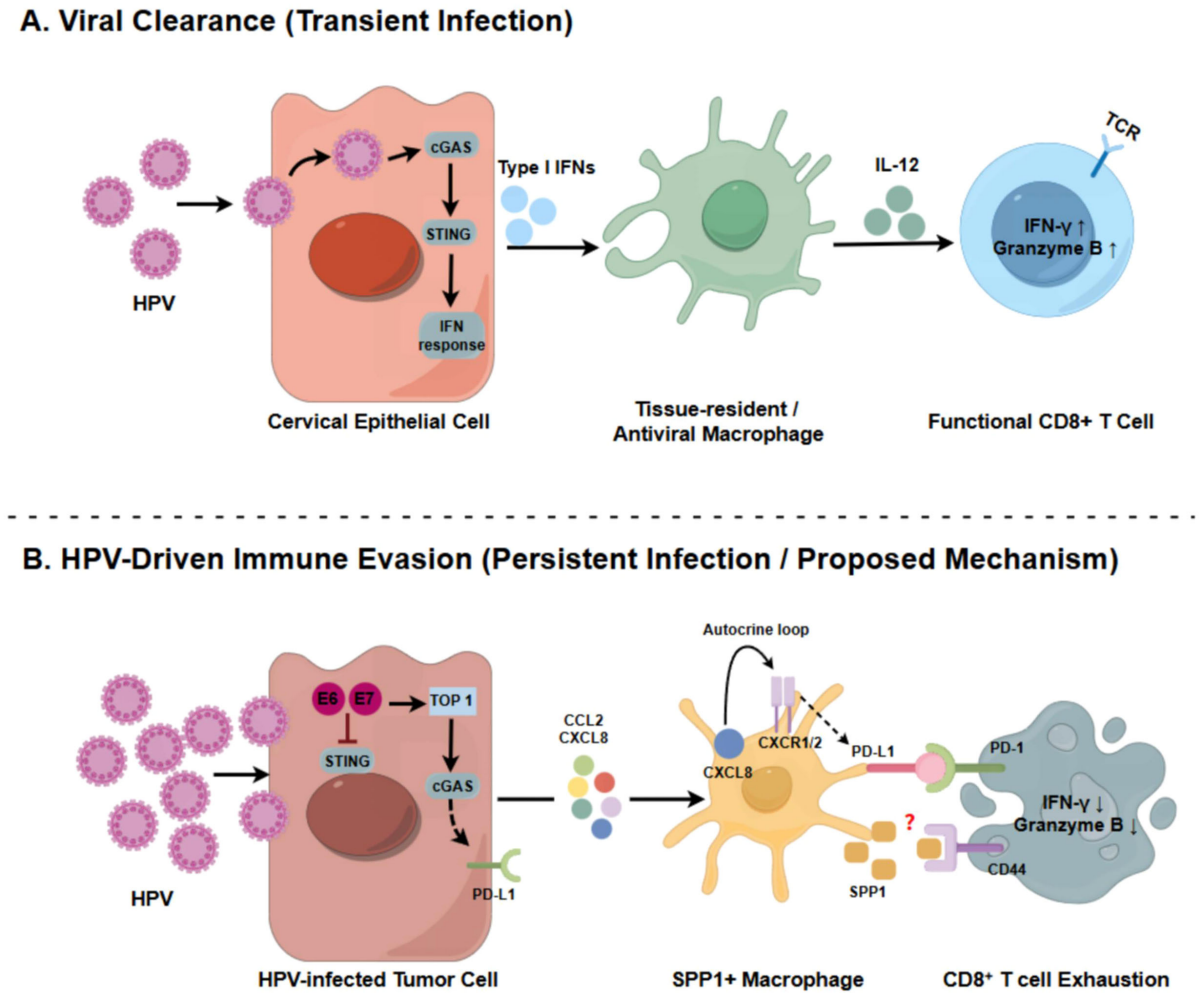
Mechanisms of viral clearance versus HPV-driven immune evasion in the cervical tumor microenvironment. **(A)** Viral clearance (Transient infection): Upon initial HPV infection, viral DNA activates the cGAS-STING pathway in cervical epithelial cells, triggering a robust Type I interferon (IFN) response. This promotes the activation of tissue-resident antiviral macrophages, which secrete IL-12 to facilitate the generation of functional CD8+ T cells characterized by high expression of IFN-γ and Granzyme B, ultimately mediating viral clearance. **(B)** HPV-Driven immune evasion (Persistent infection/Proposed mechanism): In persistent infections, HPV oncoproteins E6 and E7 abrogate the STING pathway (e.g., via TOP1 inhibition). Concurrently, the tumor cells secrete chemokines (CCL2, CXCL8) to recruit macrophages. An autocrine CXCL8 loop further solidifies the immunosuppressive phenotype of SPP1+ macrophages. Alongside PD-L1/PD-1 engagement, the interaction between SPP1 (secreted by macrophages) and its receptor CD44 on T cells is hypothesized to promote CD8+ T cell exhaustion, establishing an immune-tolerant niche for tumor progression. Note: The question mark ()? denotes that the SPP1-CD44 axis represents a candidate mechanism primarily inferred from transcriptomic data and murine models, requiring further functional validation in human cervical cancer.

Importantly, exhaustion should not be interpreted as complete functional inertness. Certain proliferative or clonally expanded T-cell populations may retain residual cytotoxic potential, but their activity can be constrained by TAMs, Tregs, CAFs, checkpoint ligands, hypoxia, nutrient deprivation, and metabolic by-products (32, 34–37, 43–45, 58, 59, 61). Thus, T-cell dysfunction is better viewed as an imbalanced operational state in which residual effector activity is progressively outweighed by inhibitory programming and spatial restriction rather than as a simple loss of killing capacity (31, 33, 35, 36, 59) (**Figure 1**).

Recent studies also suggest that T-cell dysfunction is context- and treatment-dependent. Integrated single-cell and spatial analyses have identified divergent CD8^+^ T-cell states and communication networks across distinct HPV-associated immune contexts (62). Paired pre- and post-chemoradiotherapy profiling further revealed a therapy-induced CD8^+^ T-cell senescence program, distinct from classical exhaustion, driven by ACKR2^+^ therapy-resistant tumor cells through TGF-β signaling and associated with recurrence (63). These findings indicate that exhaustion, senescence, and terminal differentiation may all limit antitumor immunity but differ in upstream drivers, reversibility, and therapeutic vulnerability (33, 36, 63).

### 3.2 CD4^+^ T-cell subsets and the suppressive Treg network

Although CD8^+^ T cells are direct mediators of antitumor cytotoxicity, CD4^+^ T cells—especially FOXP3^+^ Tregs—are critical determinants of immunosuppressive tone in the cervical tumor microenvironment (34, 35, 44, 61, 65). Single-cell atlases indicate that cervical cancer Tregs are heterogeneous. One study identified Tregs prominently marked by TNFRSF4/OX40 (35), whereas spatially resolved analyses suggested that different histological subtypes may harbor distinct CD4^+^ suppressive architectures, including ICOS^+^ Th1-like Treg features enriched in cervical adenocarcinoma (61). These findings indicate that immune suppression in cervical cancer cannot be understood solely through exhausted CD8^+^ T cells, but instead involves coordinated Treg expansion, TAM-mediated suppression, stromal restriction, and tumor-cell-derived immune evasion (32, 34, 35, 37, 43–45, 58, 61, 65).

Mechanistically, Tregs may suppress antitumor immunity through FOXP3, IL2RA, CTLA4, TIGIT, ICOS, TNFRSF4, and related regulatory pathways (34, 35, 44, 61, 65). In HPV-associated cervical cancer, the TIM-3/Galectin-9 pathway has been reported to promote Treg-mediated immunosuppression while inhibiting Th1 and CD8^+^ T-cell cytotoxic functions; blockade of this axis restored T-cell proliferation and increased IFN-γ, IL-2, granzyme B, and perforin production (35). Pathological studies across the cervical lesion continuum further suggest that FoxP3^+^ Treg frequencies increase with lesion severity (15, 59), implying that Treg-mediated suppression may be progressively established during persistent HPV infection and cervical neoplastic evolution rather than appearing only in invasive disease (11–17, 44).

Together, Tregs, suppressive TAM programs, CAF-derived stromal barriers, and checkpoint networks form a self-reinforcing suppressive circuit that can dampen effector T-cell activation, infiltration, and cytotoxicity through cytokine signaling, receptor–ligand interactions, metabolic constraints, and stromal exclusion (34, 37, 43–45, 58, 61, 65).

### 3.3 scTCR-seq reveals constrained clonal expansion and lineage skewing

If scRNA-seq defines T-cell transcriptional states, scTCR-seq links these states to clonal expansion and potential antigen-driven responses (31, 35, 36, 59, 60, 62, 63). This distinction is especially important in HPV-associated cervical cancer, where expanded clones may recognize viral antigens, tumor-associated antigens, neoantigens, unrelated inflammatory antigens, or antigens from coexisting infections (11–17, 31, 59, 60, 62). Therefore, clonal expansion should not be automatically equated with productive antitumor immunity.

Current cervical cancer studies confirm the presence of expanded T-cell clonotypes, but not all expanded clones sustain durable effector function (35, 36, 59, 60, 62). A multiomic study showed that large T-cell clones were more likely to retain cytotoxic features and may represent key antitumor effectors; nevertheless, the overall T/NK compartment displayed broad cytotoxic decline and exhaustion-associated remodeling (59). Thus, the central issue is not merely whether clonal expansion occurs, but whether expanded clones preserve effector function, access malignant epithelial compartments, and avoid suppression by TAM-, Treg-, and CAF-rich niches (34, 36, 37, 43–45, 58, 59, 61, 62).

The concept of “clonal replacement,” defined as loss or displacement of pre-existing tumor-reactive clones by newly expanded but functionally distinct clones during progression or therapy, should currently be considered a framework borrowed from other tumor types rather than a mechanism directly established in cervical cancer (31, 60). Existing TCR repertoire studies instead support a more cautious interpretation: repertoire diversity, similarity, and dynamic alterations are associated with prognosis, suggesting immune selection pressure and skewing of the antigen-recognition landscape (60). Based on available evidence, cervical cancer is best described as showing constrained clonal expansion and lineage skewing rather than definitive clonal replacement (36, 59, 60, 62, 63).

A key unresolved question is whether expanded clonotypes are positioned within tumor nests where they can contact malignant cells, or instead trapped in stromal, Treg-rich, or myeloid-rich suppressive niches (37, 43–45, 58, 61, 62). Future studies should integrate scTCR-seq, spatial transcriptomics, multiplex imaging, and HPV antigen-specific tracking to distinguish tumor-reactive clones from exhausted, senescent, or bystander clones in chronic inflammatory states (19–22, 31, 50, 59, 62, 63).

### 3.4 Spatial restriction of T-cell function

**Figure 2:**
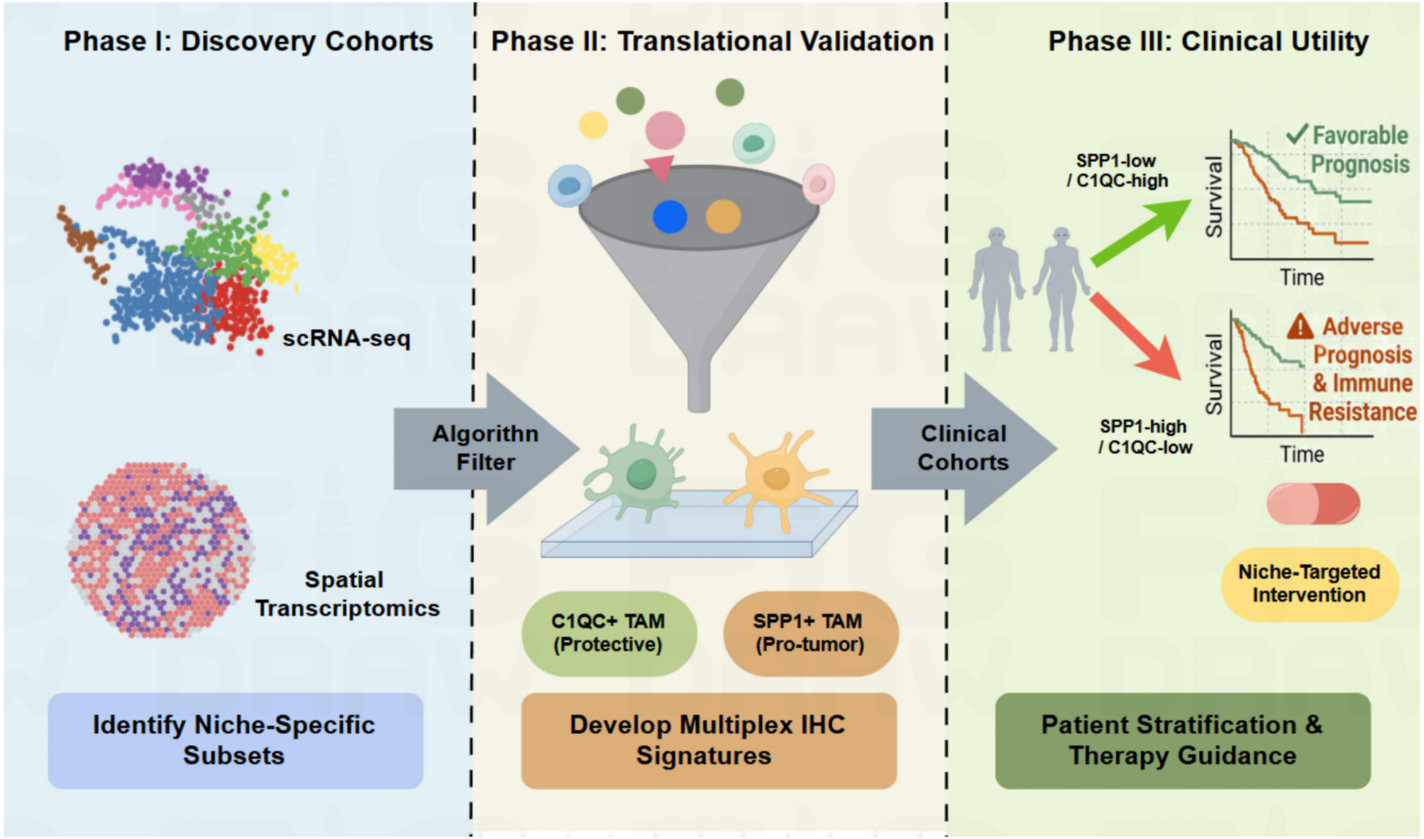
Translational pipeline: from multi-omic discovery to clinical patient stratification. A three-phase translational framework for validating macrophage-based biomarkers in HPV-associated cancers. Phase I (Discovery cohorts): High-throughput single-cell RNA sequencing (scRNA-seq) and Spatial transcriptomics are utilized to identify niche-specific macrophage subsets. Phase II (Translational validation): Through computational algorithm filtering, specific signatures (such as protective C1QC+ TAMs and pro-tumor SPP1+ TAMs) are validated at the protein level using multiplex immunohistochemistry (mIHC) on patient slides. Phase III (Clinical utility): The validated signatures serve to stratify clinical cohorts. Patients with an “SPP1-low/C1QC-high” signature exhibit a favorable prognosis. Conversely, patients with an “SPP1-high/C1QC-low” status face an adverse prognosis and immune resistance, underscoring the necessity for specific niche-targeted interventions.

Beyond transcriptional exhaustion and clonal skewing, spatial positioning is a critical determinant of whether T cells can execute antitumor immunity (19–22, 50, 61, 62). In cervical cancer, an inflamed immune phenotype does not necessarily indicate effective tumor control, because cytotoxic or clonally expanded T cells may be excluded from tumor nests, retained within fibroblast-rich stromal regions, or positioned near suppressive macrophage and Treg compartments (32, 34, 37, 43–45, 58, 61, 62). Spatial transcriptomics and multiplex imaging can therefore distinguish immune-infiltrated, immune-excluded, immune-desert, and immune-suppressed tumor regions (19–22, 38–45, 48, 50, 61, 62) (**Figure 2**).

This spatial restriction is particularly relevant for interpreting checkpoint blockade response. Tumors may contain abundant PDCD1^+^ or GZMB^+^ T cells, but if these cells are separated from malignant epithelial compartments or embedded in SPP1^+^ TAM-, CAF-, or Treg-rich niches, their effective cytotoxic access may remain limited (25, 32, 34, 37, 43–45, 56, 58, 61, 62). Conversely, localization of precursor-like or proliferative CD8^+^ T cells near tumor nests, antigen-presenting niches, or organized lymphoid aggregates may identify microenvironments with greater potential for immune reinvigoration (31, 35, 36, 59, 61, 62). Spatially resolved TCR studies will be needed to determine whether expanded clonotypes are functionally engaged with tumor cells or sequestered in suppressive neighborhoods (19–22, 50, 59, 62, 63).

Overall, single-cell, spatial, and TCR-resolved studies indicate that T-cell dysfunction in cervical cancer is best understood as a continuum of activation, exhaustion-like dysfunction, senescence, clonal skewing, and spatial restriction rather than binary functional collapse (31, 35, 36, 59–63). These states are shaped by HPV antigen persistence, tumor-intrinsic immune evasion, suppressive TAM and Treg programs, CAF-derived stromal barriers, metabolic stress, and therapy-induced remodeling (11–17, 32, 34, 37, 43–45, 58, 61–63, 65). The next challenge is to define how malignant epithelial cells, TAMs, Tregs, CAFs, and vascular-associated elements construct suppressive niches and whether these niches can be therapeutically disrupted (7, 37–45, 48, 52, 58, 61–64).

## 4 Spatial macrophage–T-cell niches and suppressive crosstalk in cervical cancer

Beyond cellular composition, a central question in the cervical cancer tumor immune microenvironment is how immune, stromal, and malignant cells are spatially arranged and how their local interactions stabilize T-cell dysfunction (19–21, 39, 41–43, 45). Recent integration of snRNA-seq, scRNA-seq, spatial transcriptomics, and multimodal pathological validation indicates that immune suppression in cervical cancer is not diffusely distributed. Instead, it is organized into structured spatial niches composed of malignant epithelial cells, TAMs, Tregs, CAFs, vascular-associated cells, and metabolic gradients (39, 41–43, 45, 48).

In this context, macrophage–T-cell crosstalk should not be interpreted only as direct TAM–T-cell contact. It also includes indirect suppression mediated by tumor cells, stromal barriers, Tregs, endothelial compartments, cytokine fields, chemokine gradients, and hypoxic or metabolically hostile regions (26–28, 43, 45, 51, 54). Therefore, the key biological question is not simply which suppressive molecules are expressed, but which cells suppress which immune programs, in which spatial niche, through which molecular axis, and under which tissue constraints.

### 4.1 From immune infiltration to spatial suppressive niche formation

Spatial localization determines whether infiltrating immune cells execute antitumor functions or become trapped within suppressive neighborhoods (19–22). In cervical squamous cell carcinoma, snRNA-seq and Stereo-seq studies showed that suppressive transcripts are not uniformly distributed. Instead, LGALS9 and IDO1 are enriched in tumor- and inflammation-associated regions, suggesting that immune suppression is spatially concentrated rather than globally homogeneous (39).

Malignant epithelial states also contribute to niche formation. Multiomic analyses indicate that epithelial-cytokeratin tumor states preferentially interact with immunosuppressive CAFs to generate TGF-β-associated immune-exclusionary microenvironments, whereas epithelial-immune states more readily engage NK and T-cell programs through interferon-responsive signaling (43). Thus, malignant cells should be viewed not only as immune targets, but also as active organizers of local immune architecture.

At the macrophage–T-cell interface, progression atlases have revealed enrichment of SPP1^+^ macrophages in invasive lesions and their spatial association with dysfunctional or exhaustion-like lymphoid compartments (32, 42, 43, 48, 51, 56). Through SPP1-centered interactions, including the candidate SPP1–CD44 axis, these macrophages may link ECM remodeling, invasion, stromal organization, and T-cell exclusion. However, because many SPP1–CD44 relationships in human cervical cancer are inferred predominantly from ligand–receptor or spatial proximity analyses, their direct causal contribution to T-cell dysfunction—while well-documented in certain murine tumor models—still requires formal functional validation in the context of human cervical malignancy.

Spatial immune organization may also differ by histology. Cervical squamous cell carcinoma tends to be relatively more immunogenic, whereas cervical adenocarcinoma more often displays stromal-rich and immune-cold features, including CAF-dominated barriers and ICOS^+^ Treg-associated suppressive architectures (41, 61). Recent spatial multi-omics further identified a CD54^+^ inflammatory CAF–ITGAL^+^ macrophage niche at the tumor–stroma interface, enriched for negative regulation of leukocyte activation, chemokine signaling, and metabolic reprogramming (45). These findings support the concept that immune exclusion in cervical cancer is actively maintained by specialized stromal–myeloid neighborhoods rather than being merely a passive consequence of desmoplasia.

### 4.2 Checkpoint and receptor–ligand circuits mediating local suppression

Within spatially defined niches, receptor–ligand interactions provide a molecular framework for local immune suppression. In cervical cancer, relevant axes extend beyond classical PD-1/PD-L1 and include co-inhibitory, adhesion-associated, macrophage-centered, and tumor-cell-mediated communication circuits (39, 42, 43, 45, 54). Nevertheless, ligand–receptor predictions should be regarded as candidate interactions unless supported by spatial co-localization, protein-level validation, and functional perturbation (**Table 3**).

**Table 3:** Major macrophage–T-cell suppressive axes in cervical cancer.

Suppressive module	Candidate axis or component	Major cellular source/target	Putative effect on T-cell immunity	Evidence status in cervical cancer
Macrophage-centered stromal remodeling	SPP1–CD44	SPP1^+^ TAMs, tumor cells, stromal cells, potentially lymphoid cells	ECM remodeling, invasion, immune exclusion, dysfunctional niche formation	Strongly inferred by single-cell/spatial studies; functional validation still needed
Co-inhibitory checkpoint signaling	PD-1/PD-L1	T cells, tumor cells, TAMs, CAF-associated niches	Reduced T-cell effector function; clinical immunotherapy relevance	Clinically established, but spatial context determines response
Treg/T-cell suppression	TIM-3/Galectin-9	T cells, Tregs, LGALS9^+^ tumor/inflammatory regions	Promotes Treg-mediated suppression and impairs Th1/CD8^+^ cytotoxicity	Supported by tissue and functional blockade evidence
Non-classical cytotoxic brake	HLA-E/NKG2A	Epithelial cells, CXCL13^+^ CD8^+^ TRM/NK-like cells	Restrains NK and CD8^+^ cytotoxic activity	Spatially suggested; requires therapeutic validation
Macrophage phagocytosis checkpoint	CD47–SIRPα	Tumor cells, macrophages	Limits phagocytosis, antigen release, and downstream T-cell activation	Mechanistic evidence for macrophage evasion; indirect T-cell relevance
Chemokine-driven recruitment	CCL2, CCL22, CXCL8	CAFs, tumor cells, macrophages, Tregs	Recruits/reprograms suppressive cells and reinforces T-cell exclusion	Supported by cervical lesion and spatial niche studies
Cytokine/stromal organization	TGF-β signaling	Tumor cells, CAFs, Tregs, stromal niches	Stromal exclusion, Treg stability, therapy-induced T-cell senescence	Supported by spatial and therapy-associated single-cell studies
Metabolic checkpointing	IDO1, lactate, hypoxia	Tumor cells, inflammatory niches, TAMs/stromal cells	Tryptophan depletion, kynurenine production, metabolic T-cell inhibition	IDO1 spatially mapped; lactate/hypoxia require more spatial validation

SPP1–CD44 is among the most consistently inferred macrophage-centered axes in cervical cancer spatial atlases (32, 42, 43, 48, 51, 56). Its potential importance lies not only in direct lymphocyte regulation, but also in connecting SPP1^+^ TAMs with ECM remodeling, stromal organization, invasion, and immune exclusion.In this proposed model—extrapolated largely from mechanistic paradigms in other solid tumors and murine systems—SPP1^+^ TAMs may promote T-cell dysfunction indirectly by constructing a physical and biochemical niche that restricts effector access to malignant epithelial compartments.

The TIM-3/Galectin-9 axis has relatively strong cervical cancer-specific support. In HPV-associated cervical cancer, elevated TIM-3 and Galectin-9 promote Treg-mediated suppression and impair Th1/CD8^+^ cytotoxic function, whereas blockade of this pathway restores T-cell proliferation and increases IFN-γ, IL-2, granzyme B, and perforin production (35). Spatial studies further show that LGALS9 is enriched in tumor- and inflammation-associated regions, supporting the local relevance of this axis (39).

Two additional non-classical checkpoints are translationally relevant but require further validation. First, HLA-E/NKG2A interactions between epithelial cells and CXCL13^+^ CD8^+^ tissue-resident memory/NK-like cytotoxic cells may provide a localized inhibitory brake on cytotoxic immunity (44). Second, CD47–SIRPα signaling enables tumor cells to evade macrophage phagocytosis; in cervical squamous carcinoma, hypoxia-induced ZEB1 strengthens this axis (54). Although CD47–SIRPα is primarily a macrophage phagocytosis checkpoint rather than a direct T-cell exhaustion pathway, it may indirectly impair T-cell immunity by limiting tumor antigen release, antigen presentation, and downstream immune activation (26, 54).

PD-1/PD-L1 remains the clinically foundational checkpoint axis in cervical cancer (2–4, 6). However, single-cell and spatial studies suggest that PD-L1 expression often operates within broader stromal, myeloid, and metabolic suppressive programs rather than as an isolated mechanism (39, 42, 43, 45). For example, CD54^+^ iCAFs may recruit and reprogram ITGAL^+^ macrophages through CCL2, which subsequently reinforces a CXCL8–PD-L1-associated suppressive circuit and CD8^+^ T-cell exclusion (45). Thus, PD-1/PD-L1 should be interpreted as one layer of a spatially organized suppressive ecosystem.

### 4.3 Cytokine, chemokine, and stromal circuits amplifying suppressive neighborhoods

Cytokine and chemokine networks extend local immune suppression across broader tissue regions (37, 39, 45, 55, 57, 65). TGF-β is a central tissue-organizing signal because it links malignant epithelial states, CAF activation, stromal exclusion, Treg stability, and therapy-associated T-cell dysfunction (43, 63). Epithelial-cytokeratin tumor states interact with immunosuppressive CAFs through TGF-β-associated programs to form immune-exclusionary microenvironments (43). Paired pre- and post-chemoradiotherapy profiling further suggests that TGF-β signaling can drive therapy-associated CD8^+^ T-cell senescence through ACKR2^+^ therapy-resistant tumor cells (63). Therefore, TGF-β should be viewed not merely as a soluble suppressive cytokine, but as a niche-organizing pathway that links stromal remodeling to impaired T-cell function.

Chemokine-mediated recruitment provides another layer of niche amplification. CCL22 expression increases along cervical lesion progression and is associated with Treg-related immune recruitment (15, 59, 65). In parallel, CD54^+^ iCAFs can deploy CCL2 to recruit ITGAL^+^ macrophages, which then reinforce local suppression through CXCL8–PD-L1-associated signaling (45). Additional studies also implicate CCL2- and CXCL8-associated programs in macrophage polarization and tumor-promoting inflammation (55, 57). Rather than representing a definitively proven sequence of events in human cervical cancer, these distinct observations across multiple studies can be synthesized into a proposed hypothetical model: a chained process in which suppressive cells are recruited by chemokine gradients, reprogrammed *in situ*, and integrated into secondary suppressive circuits involving Tregs, TAMs, CAFs, and checkpoint ligands.

Importantly, chemokines are not intrinsically suppressive. Their functional consequences depend on cellular source, receptor distribution, and tissue context. The same chemokine family may recruit effector lymphocytes in one niche but monocytes, Tregs, or suppressive macrophages in another. Therefore, chemokine signaling in cervical cancer should be interpreted spatially rather than solely by ligand expression level.

### 4.4 Metabolic suppression and hypoxic niche constraints

Metabolic rewiring further consolidates suppressive niches by limiting T-cell persistence and effector function (37, 39, 45, 66). IDO1 is a particularly relevant metabolic checkpoint in cervical cancer. Spatial transcriptomic studies map IDO1 to inflamed tumor-associated regions in cervical squamous cell carcinoma (39). Through tryptophan depletion and kynurenine production, IDO1 may link inflammatory clustering to metabolic T-cell inhibition (11, 37, 39). However, the precise cellular sources of IDO1 and their spatial relationship with dysfunctional T cells require further deconvolution and protein-level validation.

Lactate-associated stress may also contribute to immune escape. Emerging cervical cancer data suggest that lactate accumulation is associated with reduced CD8^+^ T-cell infiltration and impaired immune activity (66). However, compared with LGALS9/TIM-3, SPP1-centered interactions, and IDO1, lactate-mediated suppression remains less spatially resolved in cervical cancer. Future work should determine whether lactate-producing malignant, stromal, or TAM states overlap with exhausted, senescent, or excluded T-cell compartments.

Hypoxia provides an additional link between metabolic stress, macrophage programming, and immune evasion. In cervical squamous carcinoma, hypoxia-induced ZEB1 enhances CD47–SIRPα-mediated resistance to macrophage phagocytosis (54). Although this mechanism does not directly define T-cell exhaustion, reduced phagocytosis and impaired antigen handling may weaken downstream T-cell priming and intratumoral immune activation. Thus, hypoxic and metabolic niches may suppress T-cell immunity both directly, by limiting effector function, and indirectly, by reshaping macrophage antigen presentation and stromal organization.

Overall, metabolic suppression should be understood as a niche-level constraint rather than a single-pathway effect. IDO1 activity, lactate accumulation, hypoxia, nutrient competition, and macrophage metabolic reprogramming may collectively determine whether T cells can maintain cytotoxicity after entering the tumor microenvironment.

### 4.5 Integrated model of TAM–T-cell suppressive ecosystems

Macrophage–T-cell crosstalk in cervical cancer is best understood as a spatially organized, multi-layered suppressive ecosystem rather than a collection of isolated pathways. SPP1^+^ TAMs, ITGAL^+^ macrophages, Tregs, CAFs, malignant epithelial states, checkpoint ligands, chemokines, and metabolic constraints cooperate to generate local immune barriers (39, 41–43, 45, 66).

In this model, CAF–macrophage alliances at tumor–stroma interfaces restrict T-cell entry; LGALS9/TIM-3 and IDO1-associated programs in inflamed tumor regions convert immune activation into local dysfunction; hypoxic regions weaken macrophage phagocytosis and antigen release; and PD-1/PD-L1 acts as a clinically important but non-isolated suppressive layer embedded within broader myeloid–stromal niches. This integrated architecture helps explain why tumors with apparent T-cell infiltration may still fail to mount effective antitumor immunity.

Decoding these spatially resolved suppressive networks will be essential for transforming immune-cold or immune-excluded cervical tumors into therapeutically reprogrammable ecosystems (2, 10, 11, 43, 45). Rather than targeting T-cell exhaustion or TAM accumulation alone, future strategies may need to dismantle the spatial niches that maintain both dysfunctional T-cell states and suppressive macrophage programs.

## 5 HPV-driven rewiring of macrophage–T-cell interaction networks

The spatially organized suppressive niches described above raise an upstream question: why are macrophage–T-cell suppressive circuits so deeply embedded in cervical cancer? A central explanation is persistent infection with high-risk human papillomavirus, HPV. Unlike many solid tumors whose immune microenvironments are shaped primarily by host-intrinsic genomic and epigenomic evolution, cervical cancer develops in the setting of persistent viral infection and sustained viral oncogene activity across the lesion-to-cancer continuum (11, 17, 48). Therefore, macrophage–T-cell crosstalk in cervical cancer should not be viewed only as a late consequence of malignant transformation. Instead, it emerges progressively through chronic HPV antigen exposure, altered innate immune sensing, persistent inflammation, and gradual establishment of local immune tolerance.

Recent single-cell and spatial atlases spanning normal cervix, HPV-infected cervix, high-grade squamous intraepithelial lesions, HSILs, and invasive cervical cancer support this view. These studies suggest that HPV acts as an upstream and persistent remodeler of the cervical tumor immune microenvironment, TIME, rather than merely as a background etiological factor (39, 42, 43, 48).

### 5.1 E6/E7-mediated disruption and redirection of innate immune sensing

As a foundational premise for the mechanistic framework presented in this section, it is essential to specify that these mechanisms primarily describe HPV-associated (HPV-positive) cervical cancer, which constitutes the vast majority of cases. HPV begins to shape the cervical immune microenvironment through viral oncoproteins, particularly E6 and E7, which interfere with host innate immune recognition. Mechanistic studies and recent reviews indicate that HPV oncoproteins can interfere with cGAS–STING-related innate immune sensing and downstream antiviral signaling, thereby attenuating viral DNA sensing and type I interferon responses (14, 17). However, this effect should not be interpreted simply as a reduction in immune-cell recruitment. Rather, impaired or distorted innate sensing may alter the quality of local immune activation and influence how myeloid cells are recruited, conditioned, and functionally polarized within HPV-infected cervical lesions and tumors (11, 14, 48).

Importantly, HPV should not be described as merely “switching off” cGAS–STING signaling. Current evidence instead supports a context-dependent model in which HPV oncoproteins can attenuate canonical antiviral DNA-sensing pathways while, in some tumor-adapted settings, selected cGAS-associated inflammatory or immune-evasive outputs may be preserved or redirected (14, 17). On the one hand, E7-related mechanisms have been shown to antagonize canonical cGAS–STING-mediated antiviral signaling, which may facilitate viral persistence and immune surveillance escape (67). On the other hand, E6/E7-driven TOP1 upregulation has been linked to chronic activation of a cGAS-associated PD-L1 pathway in cervical cancer development, thereby contributing to local checkpoint induction and inflammatory immune escape (13).

These observations should be interpreted as distinct and not yet fully reconciled findings, rather than as a single linear model in which HPV uniformly suppresses or activates cGAS–STING signaling. The determinants of these divergent outputs remain incompletely defined, but may include HPV genotype, viral integration status, lesion stage, epithelial or immune-cell context, chronicity of E6/E7 expression, baseline DNA damage or replication stress, and the specific downstream branch being measured, such as type I interferon production, NF-κB-related inflammatory signaling, or PD-L1 induction (13, 14, 17).

The therapeutic implications of this axis also require cautious interpretation. Pharmacological activation of STING/TBK1 has been reported to promote targeted degradation of HPV16/18 E7 oncoproteins and suppress HPV-positive cervical cancer growth in experimental models (12). However, the generalizability of this observation to heterogeneous human cervical cancer populations, including tumors with different HPV genotypes, integration patterns, immune states, and treatment histories, remains uncertain.

Thus, in cervical cancer, cGAS–STING-related signaling is better conceptualized as a context-dependent innate immune signaling node rather than a uniformly suppressed or uniformly activated pathway. Some antiviral branches may be attenuated to permit viral persistence, whereas selected downstream outputs may, in particular tumor contexts, contribute to inflammatory immune escape, checkpoint induction, and tumor progression (13, 14). This rewiring may influence macrophage–T-cell communication. When early antiviral alarm signals are weakened or distorted, myeloid cells entering or residing within HPV-infected tissue may be less likely to sustain acute infection-clearing programs and more likely to acquire tissue-repair, tolerogenic, or protumorigenic features (11, 14, 48). In parallel, T cells may be exposed not to a transient inflammatory environment optimized for viral clearance, but to chronic antigenic stimulation combined with checkpoint signaling, metabolic stress, and suppressive myeloid–stromal cooperation (39, 42, 43, 45). Therefore, the HPV-driven cervical TIME should be viewed as a chronic infection-shaped state of immunological disequilibrium rather than as a generic tumor-suppressive milieu (11, 14, 17, 48).

### 5.2 Spatiotemporal evolution of macrophage–T-cell networks from HPV infection to invasive cancer

High-resolution atlases demonstrate that immune abnormalities in cervical carcinogenesis do not arise abruptly at the invasive stage. Instead, they accumulate progressively along the continuum from persistent HPV infection to cervical intraepithelial neoplasia, HSIL, and invasive carcinoma (39, 42, 43, 48). Integrative single-cell and spatial studies reveal continuous remodeling of cellular composition, intercellular communication, and spatial neighborhoods across disease progression. Importantly, premalignant lesions and invasive cancers may share key epithelial and immune programs, suggesting that components of the suppressive state are established before frank invasion (42, 43, 48).

At the macrophage level, this evolution is characterized by a shift from inflammatory surveillance and antigen-handling programs toward tissue-remodeling, angiogenic, and suppressive phenotypes. Across cervical cancer atlases, SPP1^+^ macrophages repeatedly emerge as a prominent TAM population associated with invasive disease, tumor–stroma communication, angiogenic remodeling, suppressive spatial niches, and adverse clinical features (32, 42, 43, 48, 51). By contrast, macrophage programs related to antigen processing, inflammatory recruitment, or tissue homeostasis appear relatively enriched in non-invasive or less advanced contexts (42, 43, 48). These observations suggest that HPV-driven progression is accompanied by a myeloid transition from immune surveillance toward matrix remodeling, immune suppression, and tumor support, with SPP1^+^ TAMs representing a late-stage amplified remodeling program in invasive disease.

The T-cell compartment evolves in parallel. As lesions progress, effector and inflammatory T-cell states become increasingly counterbalanced by exhausted, regulatory, or dysfunctional populations (32, 33, 42, 43, 48). Spatial analyses indicate that suppressive macrophage populations, including SPP1^+^ TAMs, can occupy neighborhoods adjacent to dysfunctional lymphoid compartments, supporting localized checkpoint communication and T-cell impairment (39, 42, 43, 45, 48). Thus, HPV-driven immune escape is not limited to progressive CD8^+^ T-cell exhaustion. It involves coordinated remodeling of CD4^+^ T cells, Tregs, macrophages, stromal cells, and malignant epithelial states, together with increasing spatial proximity between suppressive myeloid programs and dysfunctional T-cell states (32, 39, 42, 43, 45, 48).

Classical immunopathological studies are consistent with this model. FoxP3^+^ Treg-associated signals increase during cervical dysplasia progression, and CCL22 expression is elevated in progressive cervical dysplasia, supporting the idea that Treg-associated tolerance begins before invasive cancer is histologically established (15, 59, 65). Additional immune-tolerance mechanisms, including HLA-G-associated pathways, have also been implicated in HPV infection, cervical intraepithelial neoplasia, and cervical carcinogenesis (15, 16). Therefore, progression from HPV infection to CIN/HSIL and invasive carcinoma should not be divided into separate “viral” and “tumor” phases. Rather, it represents a continuous immunological trajectory in which persistent viral pressure gradually stabilizes suppressive spatial niches.

HPV status may also influence the dominant logic of immune communication in established cervical cancer. Although most cervical cancers are HPV-associated, HPV-negative tumors should not be assumed to share identical immune architectures or immune-evasion routes (11, 17). In HPV-positive tumors, continuous viral antigen expression, altered innate immune sensing, and viral oncoprotein activity may shape antigen-presentation context, checkpoint expression, and inflammatory signaling (13, 14, 17). By contrast, HPV-negative tumors may rely more heavily on host-intrinsic oncogenic programs and non-viral mechanisms of immune evasion. Crucially, high-resolution single-cell and spatial transcriptomic datasets detailing macrophage–T-cell organization specifically in HPV-negative cervical cancer remain severely limited. Consequently, it is not yet known whether the stepwise myeloid–T-cell crosstalk, chemokine staging, or SPP1^+^ TAM dominance observed in HPV-positive datasets are conserved in HPV-negative tumors, or if they employ distinctly uncoupled immune-evasion architectures. This distinction refines the functional significance of HPV status: HPV determines not only the presence of viral antigens, but also the routing of immune information and the cellular architectures through which local immune responses are organized (11, 17, 42, 48).

Taken together, HPV should not be reduced to a single initiating carcinogenic trigger in cervical cancer. It acts as a long-term upstream programmer of macrophage–T-cell interaction networks. By reshaping innate immune sensing, antigen-presentation context, checkpoint expression, myeloid polarization, Treg recruitment, and T-cell state transitions across the lesion continuum, persistent HPV infection progressively biases the cervical microenvironment toward a stable and self-reinforcing suppressive ecosystem (11, 13, 14, 17, 39, 42, 43, 48). This dual identity, as both a virus-associated tumor and a chronic inflammation-associated tumor, gives cervical cancer a mechanistic and therapeutic logic distinct from many other solid malignancies.

## 6 Translational paradigms: from high-dimensional atlases to clinical utility

The clinical value of single-cell and spatial profiling in cervical cancer will ultimately depend on whether atlas-derived observations can be converted into reproducible, testable, and pathology-compatible biomarkers. At present, scRNA-seq, snRNA-seq, spatial transcriptomics, and high-plex spatial proteomics remain costly, technically demanding, and insufficiently standardized for routine diagnostic implementation. Therefore, the most realistic translational strategy is not to directly introduce discovery-scale platforms into daily clinical practice, but to distill minimal and biologically informative units from these atlases and validate them using formalin-fixed paraffin-embedded (FFPE)-compatible methods, including qPCR, NanoString, immunohistochemistry, multiplex immunofluorescence, and digital pathology (43, 45, 50, 68).

Several methodological caveats should be considered before clinical translation. First, dissociation-based single-cell approaches may underrepresent fragile, rare, or matrix-embedded populations and may distort apparent cell-state abundance. Second, spatial platforms are not analytically interchangeable, particularly in FFPE tissue, because panel design, sensitivity, field-of-view selection, tissue quality, and segmentation performance can substantially affect biological readouts (39, 43, 45, 50, 68). Third, transcript-based ligand–receptor predictions should be regarded as hypothesis-generating unless supported by protein-level colocalization, spatial proximity, perturbation experiments, or functional validation (39, 43, 45). These limitations do not diminish the value of high-dimensional atlases. Rather, they define the validation standards required to convert atlas-derived insights into clinically useful tools.

### 6.1 Distilling core molecular atlases into prognostic and stratification models

From a translational perspective, one of the most important contributions of single-cell and spatial atlases is the shift from categorical “cell types” toward quantifiable “cell-state signatures.” For clinical deployment, robust biomarkers are unlikely to rely on isolated single genes. Instead, compact gene panels or spatially resolved protein signatures that reproducibly capture suppressive TAM programs, exhausted T-cell hierarchies, stromal–myeloid states, or defined immune-exclusion niches are more likely to achieve both biological interpretability and clinical robustness (39, 42, 43, 45, 51).

Cervical cancer already provides a proof of concept for this strategy. TAM-focused analyses have shown that C1QC^+^ and SPP1^+^ macrophage signatures outperform conventional M1/M2 gene sets in stratifying patients by prognosis, tumor stage, immune infiltration, and checkpoint expression (51). In this framework, patients with a C1QC^high/SPP1^low profile exhibit more favorable clinical features, whereas those with a C1QC^low/SPP1^high profile show poorer trajectories (51). This finding supports a broader conceptual shift: the clinically relevant diagnostic unit is not the oversimplified M1/M2 label, but biologically grounded myeloid programs that can be cross-validated across independent atlases, bulk transcriptomic cohorts, and pathology-based assays (42, 43, 51).

This translational framework is further reinforced by recent cervical cancer studies. Single-cell and spatial analyses repeatedly identify SPP1^+^ TAMs as a suppressive, remodeling, and clinically adverse macrophage state (32, 42, 43, 48, 51). Spatial multi-omics also places macrophage-rich stromal niches within broader suppressive ecosystems involving CAFs, chemokines, PD-L1 induction, and CD8^+^ T-cell exclusion (45). Earlier bulk RNA-based immune prognostic models further suggest that curated immune gene signatures can reflect PD-1/PD-L1 pathway activity, immune infiltration, and local immune engagement (52, 69). Together, these findings support the development of trajectory-informed and spatially informed signatures that retain biological interpretability while remaining compatible with cohort-level validation and routine pathology workflows (43, 45, 51, 52, 69).

A practical translational pipeline is therefore emerging for cervical cancer immunology: high-dimensional atlas discovery, algorithmic feature reduction, validation across bulk or real-world transcriptomic datasets, orthogonal confirmation in independent FFPE cohorts, and final conversion into clinically deployable assays such as multiplex immunofluorescence, immunohistochemistry-based scoring systems, or digital pathology classifiers (43, 45, 50, 68). In this pipeline, the goal is not to reproduce the entire atlas in the clinic, but to identify the smallest reproducible biomarker set that captures the relevant biology. Only through this reduction-and-validation strategy can atlas-derived discoveries move beyond descriptive bioinformatics and mature into candidate companion diagnostics or patient-stratification tools.

### 6.2 New targets and combinatorial strategies to overcome immune tolerance

Therapeutically, advanced cervical cancer has entered the immunotherapy era, but clinical benefit remains constrained by profound microenvironmental heterogeneity. Pembrolizumab plus chemotherapy, with or without bevacizumab, has become an important first-line option for persistent, recurrent, or metastatic cervical cancer with PD-L1-positive disease, and immune checkpoint blockade has reshaped the therapeutic landscape of advanced cervical cancer (2–4, 6, 9). In addition, although not an immune checkpoint therapy, the tissue factor-directed antibody–drug conjugate tisotumab vedotin has expanded treatment options for recurrent or metastatic disease progressing after chemotherapy (5). Contemporary clinical guidelines reflect this evolving therapeutic landscape (9, 70). However, these advances do not fully address a central translational question: which patients exhibit primary or adaptive resistance because their tumors are organized around localized suppressive ecosystems that cannot be reversed by checkpoint blockade alone? Addressing this question requires integrating the structural niche architecture revealed by single-cell and spatial atlases (39, 43, 45).

Among emerging translational directions, one particularly compelling strategy is to combine immune checkpoint inhibition with therapies targeting the stromal–myeloid circuits that sustain PD-L1 expression and T-cell suppression. Targeting the CD54^+^ iCAF–ITGAL^+^ macrophage–CXCL8–PD-L1 axis described above provides a representative example of atlas-guided combination therapy. In this model, CXCR1/2 inhibition with reparixin synergized with PD-L1 blockade, illustrating how spatial atlases can nominate interventions that dismantle suppressive stromal–myeloid niches rather than simply add agents empirically (45).

**Figure 3:**
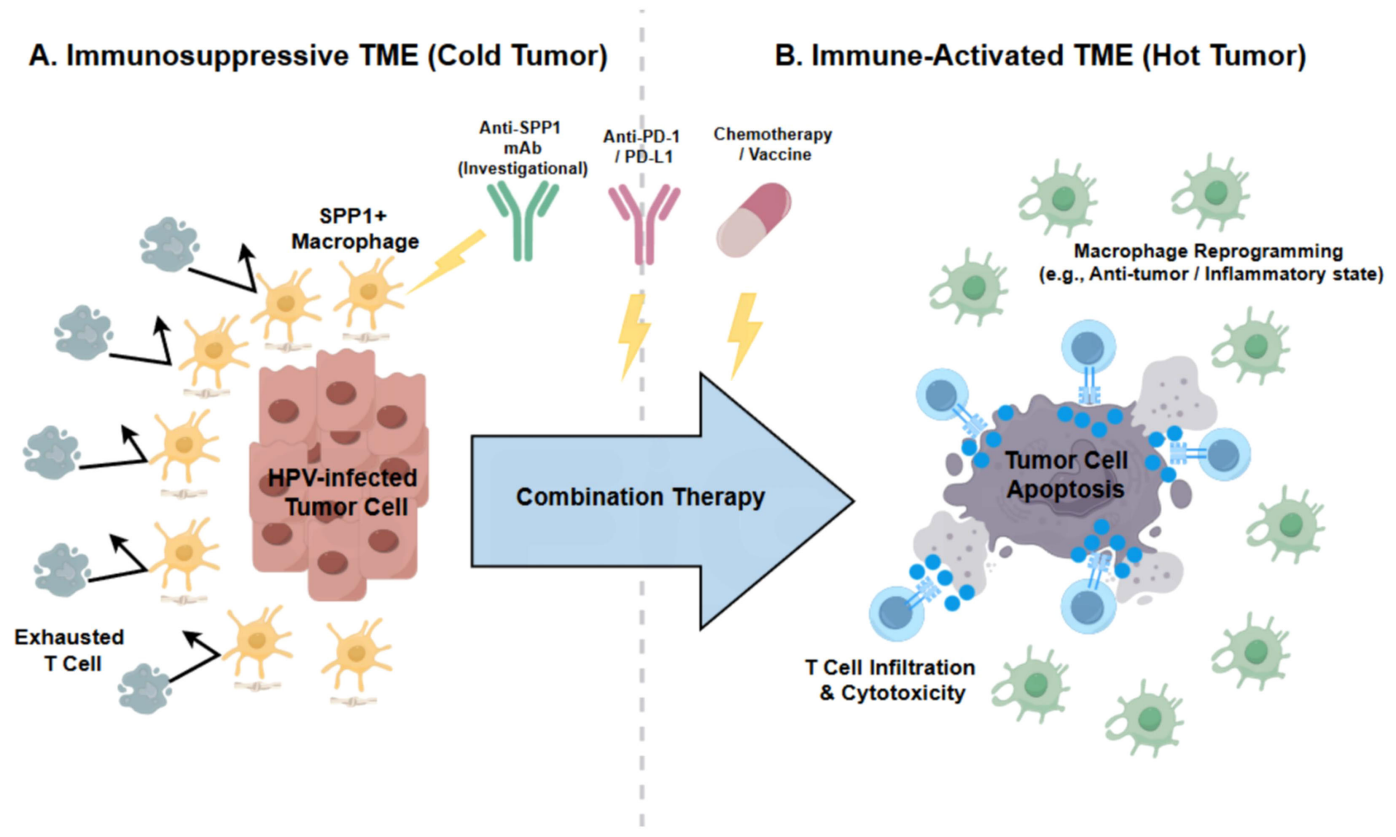
Therapeutic reprogramming: breaking the SPP1+ macrophage barrier via combination strategies. **(A)** Immunosuppressive TME (Cold tumor): In the untreated state, SPP1+ macrophages construct a dense physical and biochemical barrier (collagen/fibers) around HPV-infected tumor cells. This niche restrains functional immune infiltration, causing resident T cells to adopt an exhausted phenotype and rendering the tumor resistant to conventional therapies. **(B)** Immune-activated TME (Hot tumor): A hypothetical combination therapy—integrating investigational Anti-SPP1 therapies, immune checkpoint inhibitors (Anti-PD-1/PD-L1), and standard chemotherapy or vaccines—aims to disrupt this barrier. The intervention is proposed to drive the successful reprogramming of TAMs into an anti-tumor/inflammatory state, enabling robust infiltration of functional CD8+ T cells. This therapeutic remodeling seeks to restore potent T cell cytotoxicity, culminating in extensive tumor cell apoptosis and immune-mediated tumor regression. Note: This figure illustrates a theoretical framework; Anti-SPP1 strategies are currently investigational and require extensive validation in cervical cancer.

SPP1-associated TAM programs represent another translationally attractive target space. As discussed above, the recurrent SPP1^+^ TAM program marks a suppressive myeloid state that may be exploited for biomarker-enriched myeloid reprogramming strategies. Although direct therapeutic targeting of SPP1-centered signaling in cervical cancer remains investigational, its recurrence across independent atlases supports its use as both a candidate stratification marker and a potential entry point for macrophage-focused intervention (**Figure 3**).

A second promising direction involves targeting metabolic and innate immune circuits as sensitizers to immunotherapy rather than as isolated therapeutic alternatives. IDO1 appears as a recurrent local metabolic checkpoint in cervical squamous spatial atlases, where it may contribute to tryptophan depletion, kynurenine production, and localized T-cell dysfunction (11, 37, 39). Lactate-associated metabolic stress may also participate in immune escape and reduced CD8^+^ T-cell infiltration in cervical cancer, although its precise spatial organization remains to be clarified (37, 66). These observations suggest that metabolic barriers may help explain why tumors with apparent immune infiltration can still fail to generate effective cytotoxic immunity.

HPV-associated innate immune rewiring offers another potential therapeutic entry point. Pharmacological activation of STING/TBK1 has been reported to promote degradation of HPV16/18 E7 oncoproteins and enhance antitumor activity, suggesting that viral oncoprotein dependency and innate immune signaling may be therapeutically exploitable in HPV-positive cervical cancer (12). At the same time, HPV E6/E7-driven TOP1 upregulation and cGAS–PD-L1 activation indicate that innate immune signaling can also be hijacked to support immune escape (13). These findings underscore the need for context-aware therapeutic design. In an HPV-rewired microenvironment, PD-1/PD-L1 blockade may be insufficient if dominant myeloid state programs, metabolic checkpoints, stromal barriers, and altered innate-sensing pathways remain intact (12, 13, 39, 43, 45).

Current evidence therefore supports prioritizing biomarker-enriched combination strategies that integrate immune checkpoint blockade with interventions targeting myeloid, stromal, chemokine, metabolic, or innate viral-sensing circuits. At present, however, these strategies should be framed as translationally prioritized hypotheses rather than practice-ready therapeutic prescriptions. Their clinical value will require cross-cohort validation, FFPE-compatible biomarker development, and prospective testing in biomarker-defined patient populations. Only through this process can single-cell and spatial atlases evolve from explanatory frameworks into clinically actionable tools for precision immunotherapy in cervical cancer.

## 7 Conclusions and perspectives

Single-cell and spatial technologies have substantially reshaped our understanding of the cervical cancer immune microenvironment. Cervical cancer is no longer best viewed as a simple mixture of infiltrating immune cells, but as a spatially organized, temporally evolving, and virally programmed ecosystem. Within this ecosystem, macrophages, T cells, stromal cells, metabolic constraints, and HPV-driven immune rewiring form interconnected suppressive niches that shape immune escape and therapeutic resistance. This conceptual shift moves the field beyond cell abundance, binary macrophage polarization, or checkpoint expression alone, toward a niche-based framework in which immune function depends on cellular state, spatial localization, intercellular coupling, and disease context.

The next challenge is translational. Discovery-scale atlases are unlikely to enter routine clinical pathology directly, but they can nominate compact, reproducible, and FFPE-compatible biomarker signatures for prognostic stratification, response prediction, and biomarker-enriched trial design. Future studies should prioritize harmonized cell-state nomenclature, protein-level spatial validation, longitudinal sampling across premalignant, invasive, HPV-associated, less common HPV-independent, and treatment-exposed disease contexts, and functional testing of candidate suppressive niches. Ultimately, progress in cervical cancer immunotherapy will depend not only on intensifying checkpoint blockade, but also on identifying and dismantling the spatial immune ecosystems that sustain resistance. A niche-informed and biomarker-guided framework may therefore provide a practical path toward more precise and effective immunotherapy combinations in cervical cancer.
